# Single-cell transcriptomics of peripheral blood reveals anti-tumor systemic immunity induced by oncolytic virotherapy

**DOI:** 10.7150/thno.74075

**Published:** 2022-10-17

**Authors:** Quanyou Wu, Xiao Hu, Xiaoli Zhang, Defeng Kong, Zhenrong Yang, Guoliang Li, Zhaoru Gu, Qi Zhang, Duo Wan, Shujun Cheng, Binlei Liu, Kaitai Zhang, Wen Zhang

**Affiliations:** 1 State Key Laboratory of Molecular Oncology, Department of Etiology and Carcinogenesis, National Cancer Center/National Clinical Research Center for Cancer/Cancer Hospital, Chinese Academy of Medical Sciences and Peking Union Medical College, Beijing 100021, China.; 2 Key Laboratory of Carcinogenesis and Translational Research (Ministry of Education), Department of Pathology, Peking University Cancer Hospital & Institute, Beijing 100142, China.; 3 National “111” Center for Cellular Regulation and Molecular Pharmaceutics, Key Laboratory of Fermentation Engineering (Ministry of Education), Hubei Provincial Cooperative Innovation Center of Industrial Fermentation, College of Bioengineering, Hubei University of Technology, Wuhan 430068, China.; 4 Department of Immunology, National Cancer Center/National Clinical Research Center for Cancer/Cancer Hospital, Chinese Academy of Medical Sciences and Peking Union Medical College, Beijing 100021, China.

**Keywords:** Oncolytic virus, Single cell sequencing, Systemic immunity, Ccl5

## Abstract

**Rationale:** Oncolytic virus (OV) therapy as a cancer therapy that improves immune status makes it a favorable candidate for optimizing immunotherapy strategies. Existing studies have focused on characterizing the disturbance of the tumor microenvironment (TME) by OV therapy. However, the changes in systemic immunity induced by OV were largely ignored, which would prevent the further understanding and optimization of oncolytic viruses.

**Methods:** The HSV-2-based oncolytic virus OH2 was used to treat tumor-bearing mouse models. The peripheral blood samples were then collected for single-cell RNA sequencing (scRNA-seq). The scRNA-seq data were analyzed using Cell Ranger, Seurat, and other bioinformatics tools. Key findings were further validated by ELISA, immunohistochemistry, flow cytometry, in vivo experiments, and clinical samples.

**Results:** Our data showed that OH2 therapy effectively activated systemic immunity and induced a sustained anti-tumor immune response. One major impact of OH2 on systemic immunity was to boost Ccl5 production, which correlated with clinical response. Besides, the cytotoxic ability of peripheral cytotoxic Cd8+ T cells and mature NK cells was elevated by OH2. Further analysis revealed that the interaction of monocytes with T cells and NK cells was critical for systemic immune remodeling and activation. We also found that systemic immune responses induced by OH2 could effectively reshape the microenvironment of distant tumor lesions and inhibit their progression.

**Conclusions:** This study is the first to comprehensively characterize the effects of OV therapy on systemic immunity, which not only sheds new light on the anti-tumor mechanisms of OH2, but also contributes to the establishment of companion diagnostics for OH2 treatment and the improvement of oncolytic therapy strategies.

## Introduction

Oncolytic virus (OV) is tumor-targeting immunotherapy based on genetically engineered viruses. OV therapy has been proven to induce specific anti-tumor immune responses through interactions between viruses, tumor cells, and the immune system 1, 2. Herpes simplex virus (HSV) is one of the most studied viruses used in OV therapy. It has two strains: HSV-1 and HSV-2. Talimogene Laherparepvec (T-VEC), a representative oncolytic herpes simplex virus type 1 (oHSV1), was genetically engineered to delete the neurovirulence gene and the genes required to inhibit antigen presentation, and to insert the granulocyte-macrophage colony stimulating factor (GM-CSF) expression cassette 3, 4. T-VEC therapy has been successfully evaluated for safety and efficacy in several different cancer types 4-6. In addition, a recent clinical study also demonstrated that OH2, one type of oncolytic virus constructed based on HSV-2, was safe and showed encouraging anti-tumor activity in patients with metastatic rectal and esophageal cancers 7.

To promote the efficacy of OV therapy, viruses are often armed with genes that enhance the immune response 8. After OV injection into the tumor lesions, the cytokines released by the armed virus often stimulate immune cells in the tumor microenvironment (TME), induce local anti-tumor immunity, and improve specific cytotoxic T lymphocyte (CTL) responses 9, 10. The ability of OV therapy to turn the TME into an immunological “hot” makes it ideal for use in combination with immune checkpoint inhibitors. Therefore, existing studies have focused on analyzing the effects of OV therapy on TME 11.

However, tumor is a systemic disease that causes numerous alterations in the composition and function of the entire immune system 12. Effective natural and therapeutically induced anti-tumor immune responses occur not only in the TME but also in the peripheral immune system (systemic immunity). Therefore, investigating how OV therapy reshapes systemic immunity would shed new light on the anti-tumor mechanisms of OV therapy and help us identify peripheral immune biomarkers for the prediction of treatment response and prognosis 13. In this study, we focused on the correlation between oncolytic virus HSV-2-based OH2 therapy and systemic immunity. Based on single-cell sequencing technology and experimental validation, we conducted transverse and longitudinal analyses of peripheral systemic immunity in mouse models after OH2 treatment, detailed the perturbation of systemic immunity by OH2 therapy, and identified potential molecular markers for establishing companion diagnostics for OH2 therapeutics.

## Materials and methods

### Cell line

The mouse colon cancer cell lines CT26 and MC-38 were purchased from the National Infrastructure of Cell Line Resource (Beijing, China) and kept by our laboratory. The cells were cultured with Dulbecco's Modified Eagle medium (DMEM, Gibco) supplemented with 10% fetal bovine serum (FBS, Gibco) in a constant temperature incubator containing 5% CO_2_ at 37 °C.

### Oncolytic virus

The oncolytic virus OH2 was derived from the wild-type HSV-2-based strain HG52 as described in our previous studies 7, 14 and was provided by Binhui Biopharmaceutical Co., Ltd. (Wuhan, China).

### Animal model construction and treatment

All animal experiments were approved by the Committee on the Ethics of Animal Experiments of the National Cancer Center/Cancer Hospital, Chinese Academy of Medical Sciences (CAMS), and Peking Union Medical College. Female BALB/c mice, C57bl/6n or CB-17 SCID (6 ~ 8 weeks old) were purchased from Beijing Vital River Laboratory Animal Technology Co., Ltd. (Beijing Vital River Laboratory Animal Technology Co., Ltd.). For the single-cell sequencing experiment, the right flank of BALB/c mice was inoculated by subcutaneous injection (s.c.) with 3 × 10^5^ CT26 cells. On day 7, the tumor volume usually reached the size of 8 ~ 10 mm in diameter, and the mice were randomized into three groups: the control group (having small tumor sizes; group1), the PBS control group (having relatively large tumor sizes; group2), and the OH2 treatment group (group3). After grouping, peripheral blood of mice in the control group (n = 6) was collected through the tail vein. The other two groups were treated with 100 μL PBS or OH2 (1 × 10^6^ pfu per mouse) by intratumoral injection (i.t.), once every two days for a total of three treatments. On the 18^th^ day after tumor implantation, peripheral blood of these two groups of mice (n = 3) was collected through the tail vein for single-cell sequencing.

For other mouse experiments, both (right and left) flanks of BALB/c and CB-17 SCID mice were inoculated by subcutaneous injection (s.c.) with 3 × 10^5^ CT26 cells. In addition, 3 × 10^5^ MC-38 cells were inoculated into both flanks of C57bl/6n mice. Mice were separated into two groups on day 7: the OH2 treatment group (n = 6 for BALB/c and n = 8 for CB-17 SCID or C57bl/6n) and the PBS control group (n = 6 for BALB/c and n = 8 for CB-17 SCID or C57bl/6n). Both groups were treated with 100 μL OH2 (1 × 10^6^ pfu per mouse) or PBS by intratumoral injection into the right flank (i.t.), once every two days for a total of three treatments. The tumor volumes were measured every 2 days after treatments and were calculated with the following formula: volume = (length × width^2^)/2. Mice were sacrificed by cervical dislocation on day 14, and spleens and both flank tumor tissues were preserved for bulk RNA-sequencing, flow cytometry, and immunohistochemistry (IHC).

### Sample collection and sequencing preparation

Peripheral blood of mice was collected from the tail vein for single-cell sequencing. The tail of the mouse was held for a certain period of time so that the temperature of the tail was raised and the blood was sufficient. We cut off the tail with scissors to ensure a neat section and collected 100 μL blood with a needle containing heparin. Blood was transferred to a centrifuge tube, and 1mL red blood cell lysis buffer (Qiagen, Germany) was added and mixed. The blood was left standing at room temperature for 10 min and centrifuged at 300 g for 5 min. The cells were washed with PBS twice for subsequent single-cell sequencing experiments.

For bulk RNA sequencing, the left flank tumor tissues were divided into small pieces and submerged in 5 vol of RNAlater (Invitrogen, USA). TRIzol (Life Technologies, USA) was used for total RNA isolation according to the manufacturer's instructions. The total RNA integrity number (RIN) of each sample was measured by RNA 6000 Nano (Agilent, USA) to ensure the validity and reliability of sequencing data. All RNA samples used in this study had an OD_260/280_ greater than 1.9 and a RIN greater than 8.5.

### scRNA-seq library preparation and sequencing

The cell suspension was counted to confirm cell viability greater than 90%, and the 10 × Genomics Full Chromium platform was used for single-cell capture and barcoding. The cDNA library was prepared and sequenced by Novogene (China).

### Sequencing data processing

We employed the mouse reference dataset and Cell Ranger software to perform scRNA-seq data processing. In detail, for the Fastq file of each sample, the count module of Cell Ranger was applied to filter, align, and count unique molecular identifiers (UMIs) with default parameters. Finally, a gene expression matrix for each sample was generated. For bulk RNA-seq data, the quality of sequencing data was measured by FastQC. Then we removed adapters and low-quality reads through Trim-Galore. The gene expression profile of each sample was calculated by Salmon 15.

### Quality control, dimension reduction, and cell clustering

A Seurat pipeline was applied to perform quality control and downstream analysis of the gene expression matrix from scRNA-seq 16. High-quality cells (> 200 genes/cells, 2500 < genes/cells, < 20% mitochondrial) were kept. We applied the decontX algorithm 17 to remove ambient RNA released from lysed cells. Red cells were manually annotated and removed. In addition, red cell marker genes were also removed from the gene-expression matrix. After stringent data quality control, a total of 68816 single cells were kept for removing batch effects. Afterward, we normalized the data and detected 5000 variable genes to perform principal component analysis (PCA). The first 30 PCA components were used to cluster distinct types of cells at a resolution of 0.5. The UMAP (Uniform Manifold Approximation and Projection) method was applied to visualize different clusters. Canonical gene markers of individual immune cell types were applied to annotate each cell cluster. Differentially expressed genes (DEGs) of the same cell clusters in different groups and marker genes of each cluster were calculated using the “FindMarkers” function and the “FindConservedMarkers” function with default thresholds, respectively.

### Over-representation analysis and module scores

We used the R package clusterProfiler 18 to perform Gene Ontology (GO) enrichment analyses for the DEGs of each cluster. The union of differentially expressed genes between group1 and group2, and between group2 and group3 returned from the GO enrichment analysis of interested pathways were selected to calculate the pathway module scores within each immune cell type. The pathway module score was identified as the percentage of counts originating from the set of selected genes in each cell.

### Measuring pathway activities by AUCell

The AUCell algorithm 19 was applied to measure the score of pathway activities for each cell and map these scores into a UMAP. In detail, the AUCell_buildRankings function was first employed to calculate gene expression rankings in each cell based on an expression matrix. Then we retrieved the pathway's gene sets from the GO database, which were used to evaluate the pathway activity in each cell by the AUCell_calcAUC function. This function scores the area-under-the-curve (AUC) values based on gene expression rankings.

### Cell interaction analysis

CellPhoneDB 20 is a database of curated ligands, receptors, and their interactions. The command-line tool “cellphonedb” which employs the CellPhoneDB database was applied to interrogate the cell-cell interactions based on ligand-receptor pairs between two cell types. Ligand-receptor pairs were further filtered according to their significance in cell-type specificity and expression level. The strength of the interaction between cell types was determined by the number of significant ligand-receptor pairs and visualized by the ComplexHeatmap 21 implemented in R.

### Measurement of the tumor immune microenvironment

CIBERSORT 22 was applied to measure the infiltration levels of 22 types of immune cells. The R package “GSVA” 23 was employed to conduct single-sample gene-set enrichment analyses to evaluate the activity of 17 key immune pathways retrieved from ImmPort 24. We also measured the expression of key immunomodulators that were collected from one previous study 25.

### Flow cytometric analysis of splenic T cells

Splenic lymphocytes were harvested by gradient centrifugation using the lymphocyte separation medium (Dakewe Biotech, China) at room temperature and washed twice with PBS. To determine cytokine expression, cells were stimulated with phorbol12-myristate 13-acetate (PMA)/ionomycin and monensin (Multiscience, China) for 6 h. For flow cytometric analysis, cells were suspended in staining buffer and incubated for 15 min at room temperature by labeling APC/Cy7 anti-mouse CD3 (Clone: 17A2, Biolegend, USA), Brilliant Violet 421^TM^ anti-mouse CD8a (Clone: 53-67, Biolegend, USA), and PE anti-mouse CCL5 (Clone: 2E9, Biolegend, USA). The cells were finally resuspended in 500 μL of PBS and subjected to flow cytometry (BD LSR II).

### Immunohistochemistry

For immunohistochemistry, both flank tumors were dissected from the immunocompetent mice and formalin-fixed and paraffin-embedded (FFPE) on day 14 after grouping. Four-micrometer-thick tissue sections were prepared and used for IHC staining. IHC was carried out according to a standard protocol. The following antibodies were used for the IHC experiment: CD8 antibody (Affinity, AF5126, China), CD16 (Affinity, DF7007, China), and F4/80 (Abcam, ab100790, USA).

### In vivo induced CTL and CTL assay

After the final treatment of immunocompetent tumor-bearing mice, spleens (n = 3 per group) were harvested. Splenic lymphocytes were isolated using the lymphocyte separation medium (Dakewe Biotech, China) at room temperature and washed twice with PBS. CT26 or MC38 cells were labeled with 0.5 mM/L CFSE for 8 min at 37 °C. After termination, the cells were collected and adjusted to 4 × 10^5^ cells/mL. Then, lymphocyte effector cells and target cells were mixed at effector/target cell (E : T) ratios of 25 : 1, 50 : 1, and 100 : 1. After incubation at 37 °C and 5% CO_2_ for 4 h, cells were harvested and labeled with 1 mg/mL PI for 5 min at room temperature, subjected to flow cytometry (LSR II, BD).

### ELISA

For cytokine analysis, 7 days after the final treatment of immunocompetent tumor-bearing mice (n = 5 per group), splenic lymphocytes were isolated using lymphocyte separation medium (Dakewe Biotech, China) at room temperature and washed twice with PBS. Splenic lymphocytes were adjusted to 1 × 10^7^ cells/mL and co-cultured with 1 × 10^6^ CT-26 or MC38 cells in RPMI with 10% FCS in a 6-well plate for 48 h. Granzyme B, TNF-α, IFN-γ, and RANTES (Ccl5) in the supernatant were analyzed by ELISA (Jingmei Biotechnology, China). For peripheral blood, the serum was performed according to the CCL5 kit instructions (Jingmei Biotechnology, China).

### Blood sampling

Collection and analysis of the clinical samples were approved by the Ethics Committee of the Cancer Institute and Hospital of the Chinese Academy of Medical Sciences. Peripheral blood was collected and coagulated at room temperature for 10 ~ 20 min and centrifuged for about 20 min (2000-3000 RPM). The supernatant was carefully collected for ELISA detection.

### Statistical analysis

All statistical tests (two-sided) and graphs drawn in this research were performed using R software. Data are expressed as the mean ± S.E.M. In addition, unless indicated otherwise, P value less than 0.05 was considered as statistically significance (*p < 0.05, **p < 0.01, ***p < 0.001, and ****p < 0.0001). All experiments were repeated in triplicate unless otherwise stated. Statistical analyses were performed using GraphPad Prism software version 8 (GraphPad Software), and statistical significance was defined as p < 0.05. Flow cytometry data were analyzed using FlowJo and compared using a two-tailed, unpaired Student's t-test.

## Results

### The overall effects of OH2 therapy on the systemic immune cells

**Figure 1:**
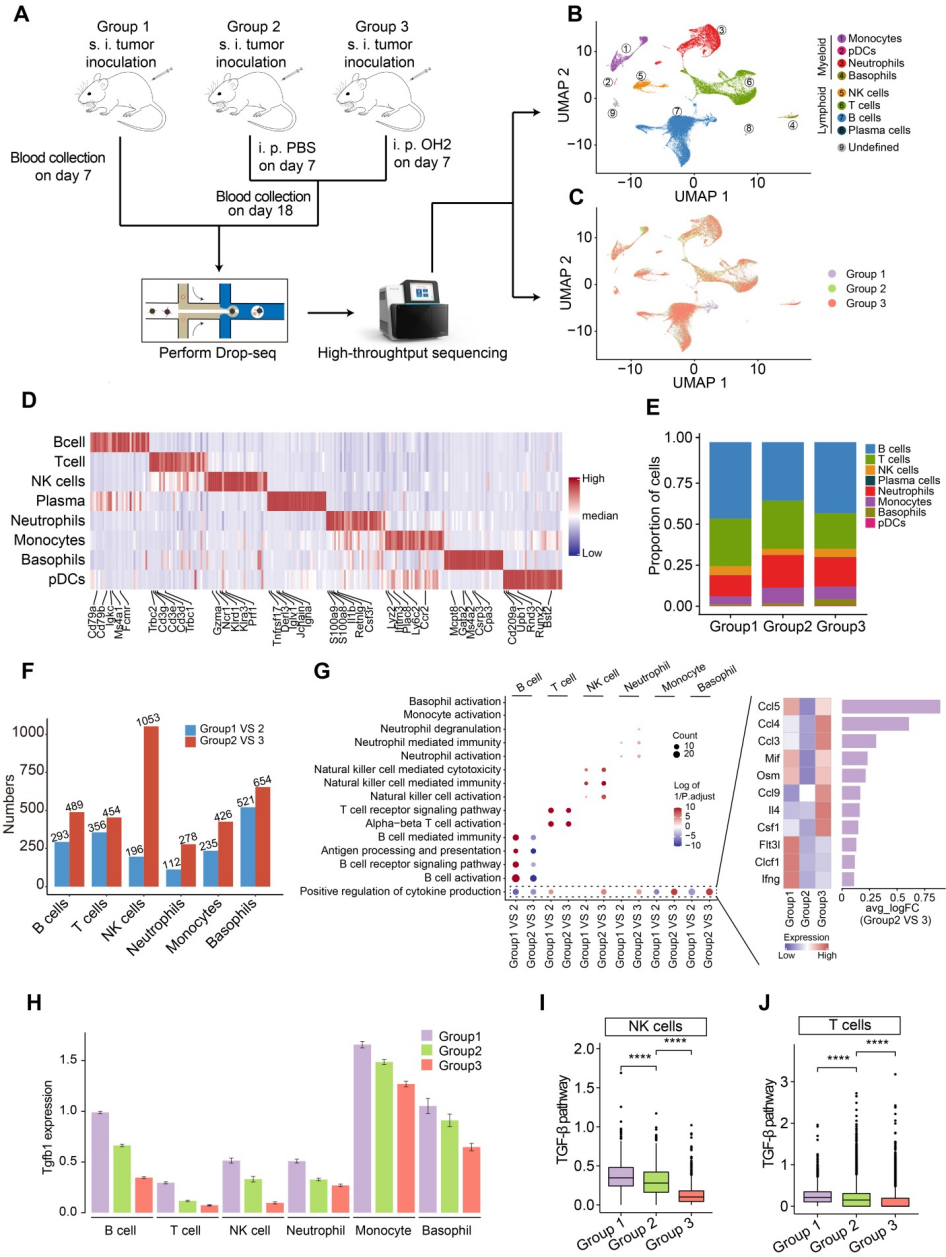
**scRNA-seq experiment and data analysis.** (A) A schematic diagram showing the overall experimental design in this study. s.c., subcutaneous injection, i.t., intratumoral injection. (B-C) UMAP plots represent the clusters (B) and groups (C) of peripheral immune cells from 12 mice. (D) A heatmap representing scaled expression values of the top 30 genes defining each type of immune cell. (E) The composition of immune cells in the peripheral blood from three experimental groups. (F) The number of differentially expressed genes in each type of immune cell caused by increased tumor burden (blue bars) and by OH2 (red bars). (G) Left panel showing the GO terms enriched by the differentially expressed genes caused by increased tumor burden and by OH2. Red and blue dots indicate that the corresponding GO terms were enriched by upregulated genes and downregulated genes, respectively. Right panel showing the mean expression of cytokines upregulated in group 3. (H) The Tgfb1 expression level of each immune cell type from three experimental groups. (I-J) Box plots representing the activity of the TGF-β pathway in NK cells (I) and in T cells (J) from three experimental groups, respectively.

To investigate the systemic immunity profiles under a tumor-bearing status and the immune-regulatory effects of OH2, we performed scRNA-seq analyses based on a total of 12 peripheral blood samples from mice classified into three groups. Group 1 contained 6 blood samples collected on the 7^th^ day after tumor inoculation in mice. Group 2 contained 3 blood samples collected on the 18^th^ day after tumor inoculation in mice, and PBS was injected into the tumor site of this group on the 7^th^ day after tumor implantation. Group3 contained 3 blood samples collected on the 18^th^ day after tumor inoculation in mice, and OH2 was injected into the tumor site of this group on the 7^th^ day after tumor implantation (Figure 1A). All white blood cells in these blood samples were retained and transformed into barcoded scRNA-seq libraries according to 10X Genomics protocols. Initial quality control and data processing of raw sequencing data were performed using Cell Ranger software.

Based on the expression of canonical markers and other genes specifically upregulated in each cell, we analyzed the distribution of immune cells and clustered these cells into lymphoid cell lineages, including T cells, B cells, NK cells, and plasma cells, and myeloid cell lineages, including monocytes, neutrophils, basophils and plasmacytoid dendritic cells (pDCs) (Figure 1B-D; Table S1). Expression values in each cell positioned in a UMAP were shown in Figure S1.

We found that the proportion of myeloid cells, especially neutrophils and monocytes, expanded with increased tumor burden (group2 VS group1) (Figure 1E), which was concordant with previous findings suggesting that the perturbations of systemic immunity induced by tumor burden are characteristic of inflation of neutrophils and monocytes in the peripheral blood 13. After the injection of OH2, the proportion of myeloid cells and lymphoid cells remained constant, with B cells increasing and T cells decreasing slightly (group3 VS group2) (Figure 1E). We further noticed that the perturbation of gene expression in peripheral immune cells caused by OH2 (group3 VS group2) was higher than that caused by increased tumor burden (group 2 VS group1) (Figure 1F). These results indicated that although OH2 had lower effects on the constituent ratio of peripheral immune cells, it exerted larger impacts on the gene expression profiles of immune cells compared to the increased tumor burden. Among all immune cells, the gene expression profiles of NK cells were most affected by OH2, which significantly altered the expression level of 1053 genes (Figure 1F). To further investigate the effects of OH2 and tumor burden on peripheral immune cells, we performed GO enrichment analysis on differentially expressed genes caused by these two purturbagens (Figure 1G). As a result, B cells, T cells, and NK cells were activated by increased tumor burden. OH2 decreased B cell activation but further significantly activated T cells and NK cells. Although OH2 played little role in the activation of myeloid cell lineages (neutrophils, monocytes, and basophils), it significantly stimulated biological pathways that participate in the positive regulation of cytokine production in these myeloid cells and NK cells. Among the increased cytokines induced by OH2, Ccl5 ranked first, followed by Ccl4 and Ccl3, suggesting that these cytokines may be critical factors mediating the perturbation of systemic immunity caused by OH2 (Figure 1G). Because previous studies reported that the TGF-β pathway was a core pathway that inhibits NK cell and T cell activities, we paid special attention to this pathway and found that Tgfb1, a key ligand initiating the TGF-β pathway, was downregulated in all types of peripheral immune cells when the tumor burden increased and OH2 was further injected (Figure 1H). Consistently, the TGF-β pathway was suppressed in NK cells and T cells after the injection of OH2 (Figure 1I-J), suggesting that downregulation of the TGF-β pathway mediated the activation of NK and T cells by OH2.

### OH2 therapy induces changes in immune responses mediated by T cells and NK cells

**Figure 2:**
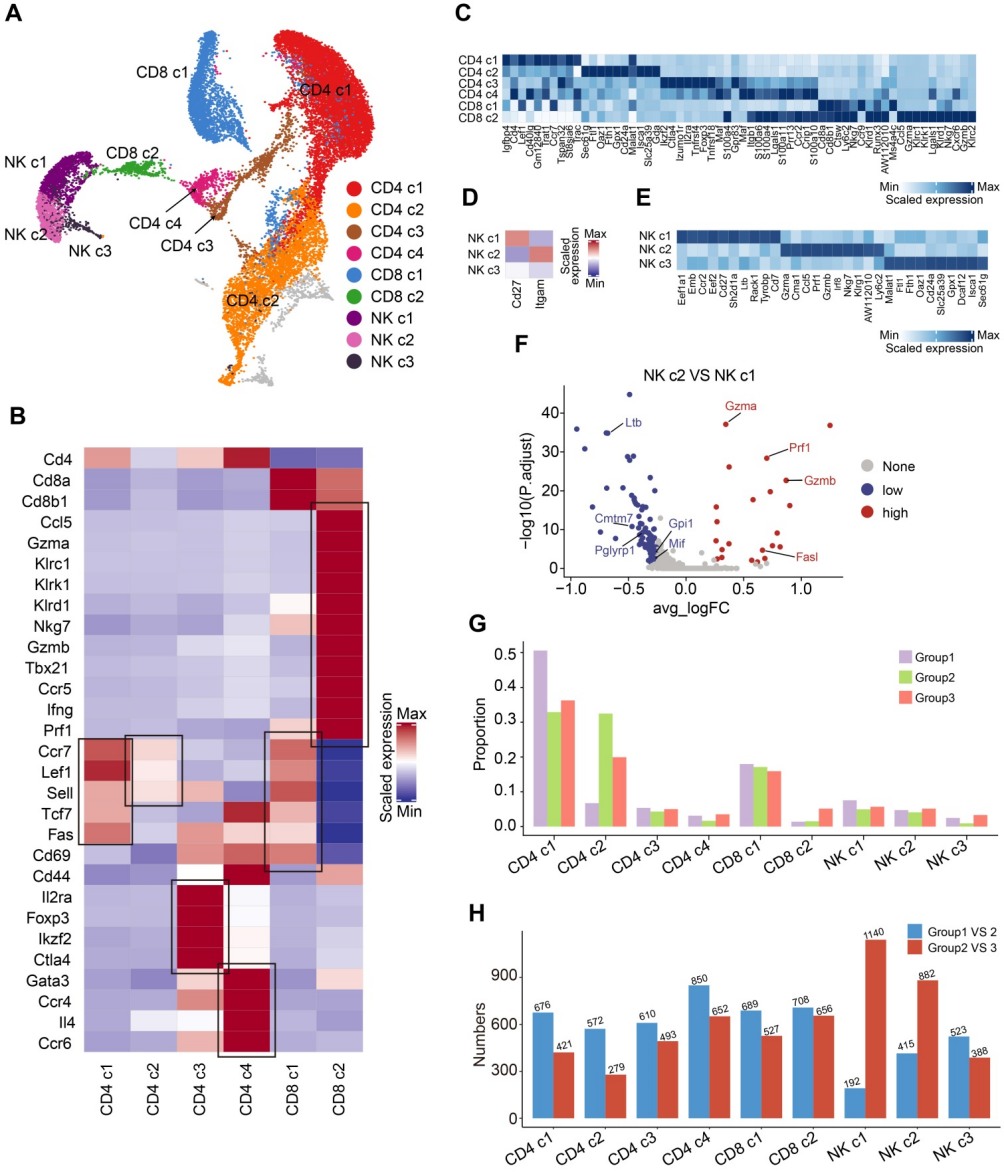
**Compositional changes of the T cells and NK cells.** (A) A UMAP displaying the subclusters of T cells and NK cells. (B) A heatmap representing the scaled average expression of canonical T cell markers in each T cell subcluster. (C) A heatmap representing scaled expression values of the top 10 genes defining each of the T cell subclusters. (D) A heatmap showing the scaled average expression of canonical NK cell markers in each NK cell subcluster. (E) A heatmap showing scaled expression values of the top 10 genes defining each of the NK cell subclusters. (F) A volcano plot representing the differentially expressed genes between NK c2 and NK c1. (G) The composition of T and NK cell subclusters in the peripheral blood from three experimental groups. (H) The number of differentially expressed genes in each T and NK cell subcluster caused by increased tumor burden (blue bars) and by OH2 (red bards).

As mentioned above, we discovered that OH2 mainly activated peripheral T cells and NK cells. To investigate the effects of OH2 on peripheral T cells and NK cells more deeply, we subgrouped T and NK lymphocytes into 9 subsets based on canonical markers (Figure 2A). Cd4^+^ T cells highly expressed Cd4 and were subdivided into four clusters: central memory Cd4^+^ T cells (Cd4 c1), which expressed high levels of Ccr7, Sell, and Fas; naïve Cd4^+^ T cells (Cd4 c2), which expressed high levels of Ccr7, Lef1, and Sell, but low levels of Fas and Cd44; regulatory Cd4^+^ T cells (Cd4 c3), which were characteristic of high levels of Il2ra and Foxp3; and Th2 cells (Cd4 c4), which highly expressed Gata3 and Il4. Cd8^+^ T cells expressed high levels of Cd8a and Cd8b and were separated into two subclusters: central memory Cd8^+^ T cells (Cd8 c1), which highly expressed Ccr7, Sell, and Fas, and cytotoxic Cd8^+^ T cells (Cd8 c2), which expressed high levels of Gzmb and Prf1 (Figure 2B-C). Based on canonical markers, we further subdivided NK cells into three subclusters: Cd27^+^ Itgam^-^ NK cells (NK c1), Cd27^-^ Itgam^+^ NK cells (NK c2), and Cd27^-^ Itgam^-^ NK cells (NK c3) (Figure 2D-E). We observed that NK c1 produced larger amounts of cytokines while NK c2 had increased cytotoxicity against target cells (Figure 2F), which was consistent with previous reports 26. NK c3 was defined as the immature NK cells.

The composition of the peripheral T and NK cell subclusters varied with increased tumor burden and the injection of OH2. The most obvious perturbation induced by increased tumor burden was the downregulation of Cd4 c1 and upregulation of Cd4 c2, suggesting that tumor burden stimulated the production of naive Cd4^+^ T cells that were released in peripheral blood. Although OH2 had fewer effects on the proportion of T and NK cell subclusters, it significantly boosted the production of cytotoxic Cd8^+^ T cells (Cd8 c2) and slightly increased the proportion of mature NK cells (NK c1 and NK c2) (Figure 2G). Furthermore, among all T and NK cell subclusters, OH2 had the most prominent effects on the gene expression profiles of mature NK cells (NK c1 and NK c2), followed by cytotoxic Cd8+ T cells (Cd8 c2) (Figure 2H).

**Figure 3:**
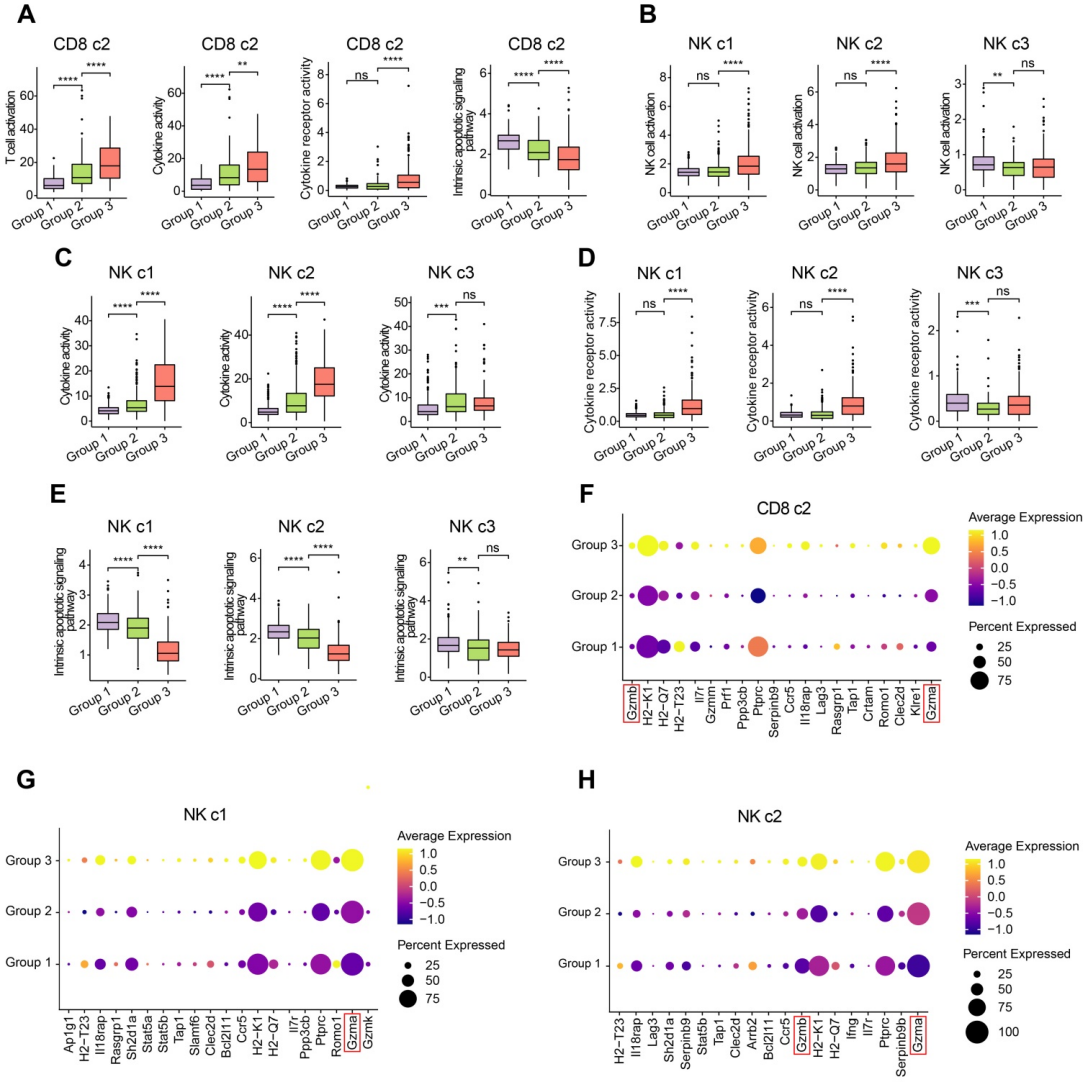
**Characterization of the T cells and NK cells.** (A) Box plot showing the degree of T cell activation, cytokine activity, cytokine receptor activity, and intrinsic apoptotic signaling pathway in CD8 c2 from three experimental groups. (B-E) Box plot representing the degree of NK cell activation (B), cytokine activity (C), cytokine receptor activity (D), and intrinsic apoptotic signaling pathway (E) in NK cell subclusters from three experimental groups. (F-H) Dot plot showing the key cytotoxicity-related genes of CD8 c2 (F), NK c1 (G), and NK c2 (H) upregulated in group 3.

To investigate the functional impacts of OH2 on NK cells and cytotoxic Cd8^+^ T cells more deeply, we characterized and compared these cells among the three experimental groups. Interestingly, we found that OH2 significantly promoted cell activation, cytokine activity, and cytokine receptor activity while suppressing the intrinsic apoptotic signaling pathway of cytotoxic Cd8^+^ T cells (Figure 3A). Similarly, the activation, cytokine activity, and cytokine receptor activity of mature NK cells (NK c1 and NK c2) were upregulated by OH2 while the intrinsic apoptotic signaling pathway of these cells was downregulated. OH2 had no significant impact on these biological processes and pathways in immature NK cells (Figure 3B-E). We further uncovered that among all cytokines, Ccl5 was upregulated most prominently in mature NK cells and cytotoxic Cd8^+^ T cells after OH2 injection (Figure S2), suggesting that Ccl5 might play a critical role in the systemic immunity remodeled by OH2. Since one of the most important characteristics of NK cells and cytotoxic Cd8^+^ T cells is their ability to lyse specific target cells, we focused on the expression perturbation of cytotoxicity-mediated genes induced by OH2 in these cells. As a result, key granzyme genes, such as Gzma and Gzmb in mature NK cells and cytotoxic Cd8^+^ T cells were significantly upregulated after OH2 injection (Figure 3F-H). All these results indicated that one major impact of OH2 on systemic immunity was to boost Ccl5 production and the cytotoxic ability of peripheral mature NK cells and cytotoxic Cd8^+^ T cells.

### OH2 therapy activated the inflammatory response of peripheral monocytes

**Figure 4:**
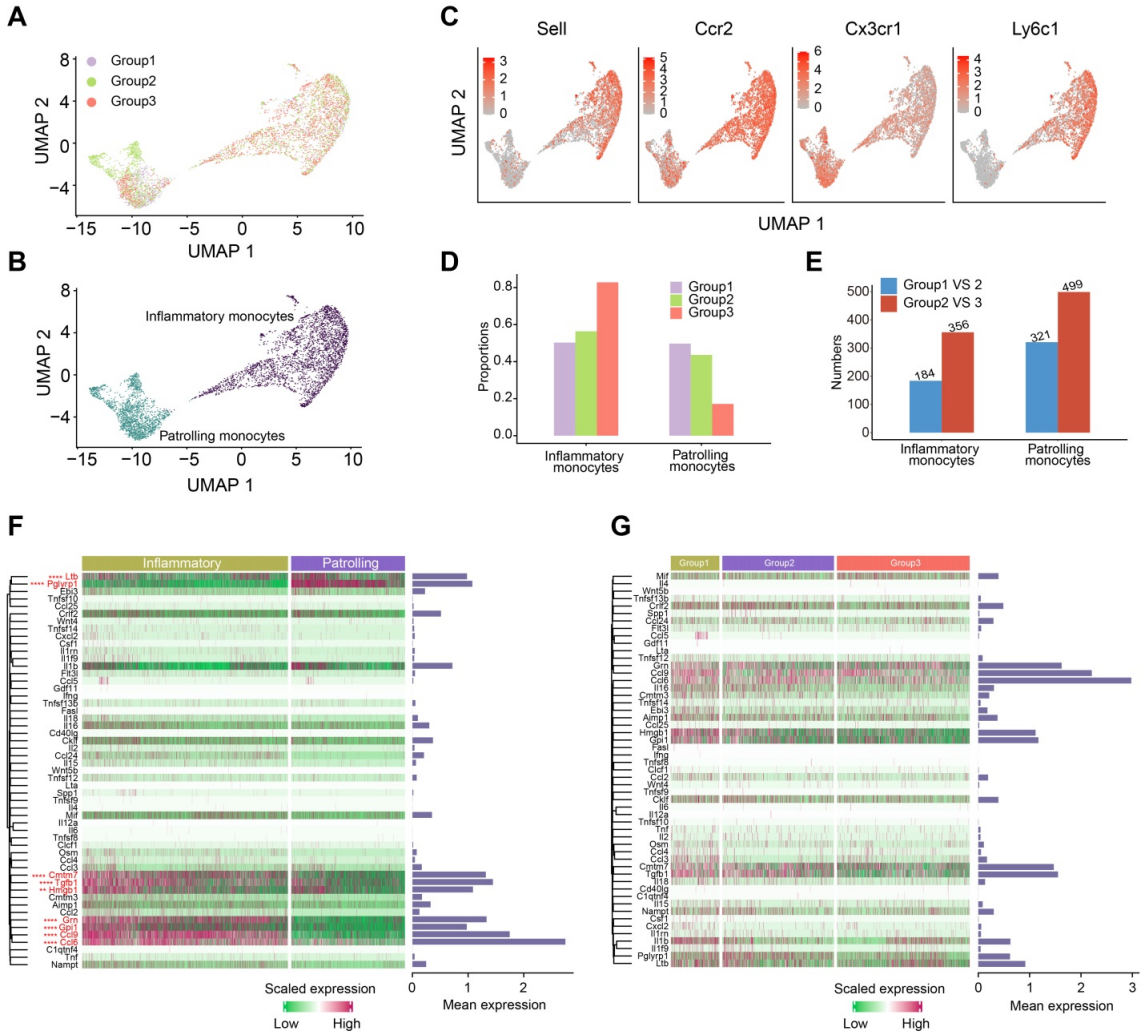
**Characterization of the monocytes.** (A-B) UMAP plots representing the groups (A) and subclusters (B) of monocytes in the peripheral blood derived from 12 mice. (C) UMAP plots showing the expression values of canonical marker genes of monocyte subclusters. (D) The composition of monocyte subclusters in the peripheral blood from three experimental groups. (E) The number of differentially expressed genes in each monocyte subcluster caused by increased tumor burden (blue bars) and by OH2 (red bars). (F) A heatmap showing the levels of cytokines produced by inflammatory monocytes and patrolling monocytes. Bars represent the mean expression of each cytokine in monocytes. (G) A heatmap showing the levels of cytokines produced by inflammatory monocytes from three experimental groups. Bars represent the mean expression of cytokines in inflammatory monocytes.

One of the most well-known functions of monocytes is the production of cytokines, which were key functional components of systemic immunity. Thus, we paid special attention to the perturbation of monocytes induced by OH2. Monocytes were classified into two subclusters: inflammatory monocytes and patrolling monocytes (Figure 4A-B). Inflammatory monocytes expressed relatively high levels of Sell, Ccr2, Ly6c1, and low levels of Cx3cr1, while patrolling monocytes expressed the opposite, with low levels of Sell, Ccr2, Ly6c1, and high levels of Cx3cr1 (Figure 4C). Interestingly, increased tumor burden had little impact on the composition of monocyte subclusters, while OH2 significantly increased the proportion of inflammatory monocytes and reduced patrolling monocytes (Figure 4D). Compared to the tumor burden, OH2 also exerted a larger impact on the gene expression profiles of monocytes (Figure 4E). We investigated the cytokine production of monocytes in particular and found that inflammatory monocytes produced larger amounts of cytokines than patrolling monocytes (Figure 4F). Focusing on inflammatory monocytes, we noticed that OH2 did not stimulate cytokine production in these cells (Figure 4G). Thus, these results suggested that the effects of OH2 on peripheral monocytes were promoting inflammatory monocyte ratios but not increasing cytokine production of each inflammatory monocyte. These insights explained why in Figure 1G, OH2 did not activate monocytes but significantly stimulated pathways that participate in the positive regulation of cytokine production in monocytes.

### OH2 had little influence on the functional characteristics of peripheral B cells and neutrophils

**Figure 5:**
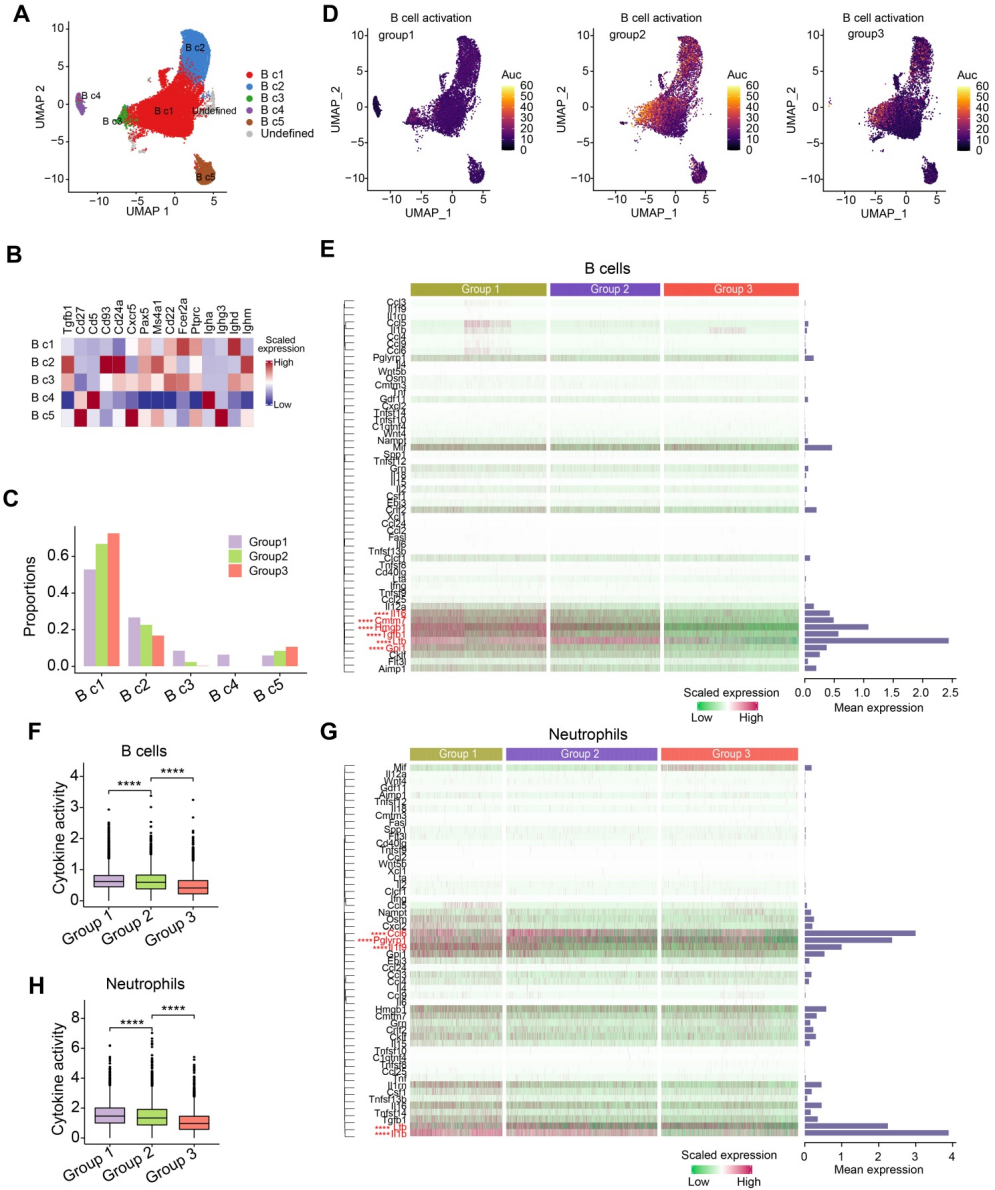
**Composition and characterization of B cells and neutrophils.** (A) A UMAP plot representing the subclusters of B cells in the peripheral blood. (B) A heatmap representing the scaled average expression of canonical B cell markers. (C) The composition of B cell subclusters in the peripheral blood from three experimental groups. (D) UMAPs represent the degree of B cell activation measured by the AUCell algorithm. (E) A heatmap showing the levels of cytokines produced by B cells. Bars represent the mean expression of each cytokine in B cells. (F) The degree of cytokine activity in B cells from three experimental groups. (G) Heatmap showing the levels of cytokines produced by neutrophils. Bars represent the mean expression of each cytokine in neutrophils. (H) A box plot representing the degree of cytokine activity in neutrophils from three experimental groups.

Since B cells and neutrophils accounted for the majority of immune cells in the peripheral blood of mice (Figure 1B), we further investigated how tumor burden and OH2 affected these immune cells. We separated B cells into five subclusters based on canonical markers: follicular B cells (naive B cells, B c1), which expressed high levels of Ptprc, Pax5 and low levels of Ighm; transitional B cells (T1, B c2), which expressed high levels of Cd24a, Ighm and low levels of Ighd and Fcer2a; regulatory B cells (B c3), which highly expressed Ighm, Ighd, and Fcer2a; IgA^+^ memory B cells (B c4), which expressed high levels of Igha and low levels of Ighd; IgG^+^ memory B cells (B c5), which expressed high levels of Ighg3 and low levels of Ighd (Figure 5A-B). We noticed that the most obvious impact of tumor burden and OH2 on the composition of B cell subclusters was increasing the proportion of naive B cells (B c1) (Figure 5C). We further evaluated the activation degree of each B cell type by the AUCell algorithm and found that the tumor burden activated B cells, especially some naive B cells, while OH2 suppressed B cell activation (Figure 5D). This result was consistent with the GO enrichment results exhibited in Figure 1G. Focusing on the cytokine production of B cells, we revealed that OH2 suppressed the expression of certain cytokines in B cells (Figure 5E-F). For neutrophils, OH2 also downregulated cytokine activity (Figure 5G-H). These results suggested that the perturbation of B cells and neutrophils induced by OH2 may have little contribution to the anti-tumor systemic immune responses.

### OH2 therapy changed interactions among peripheral immune cells

**Figure 6:**
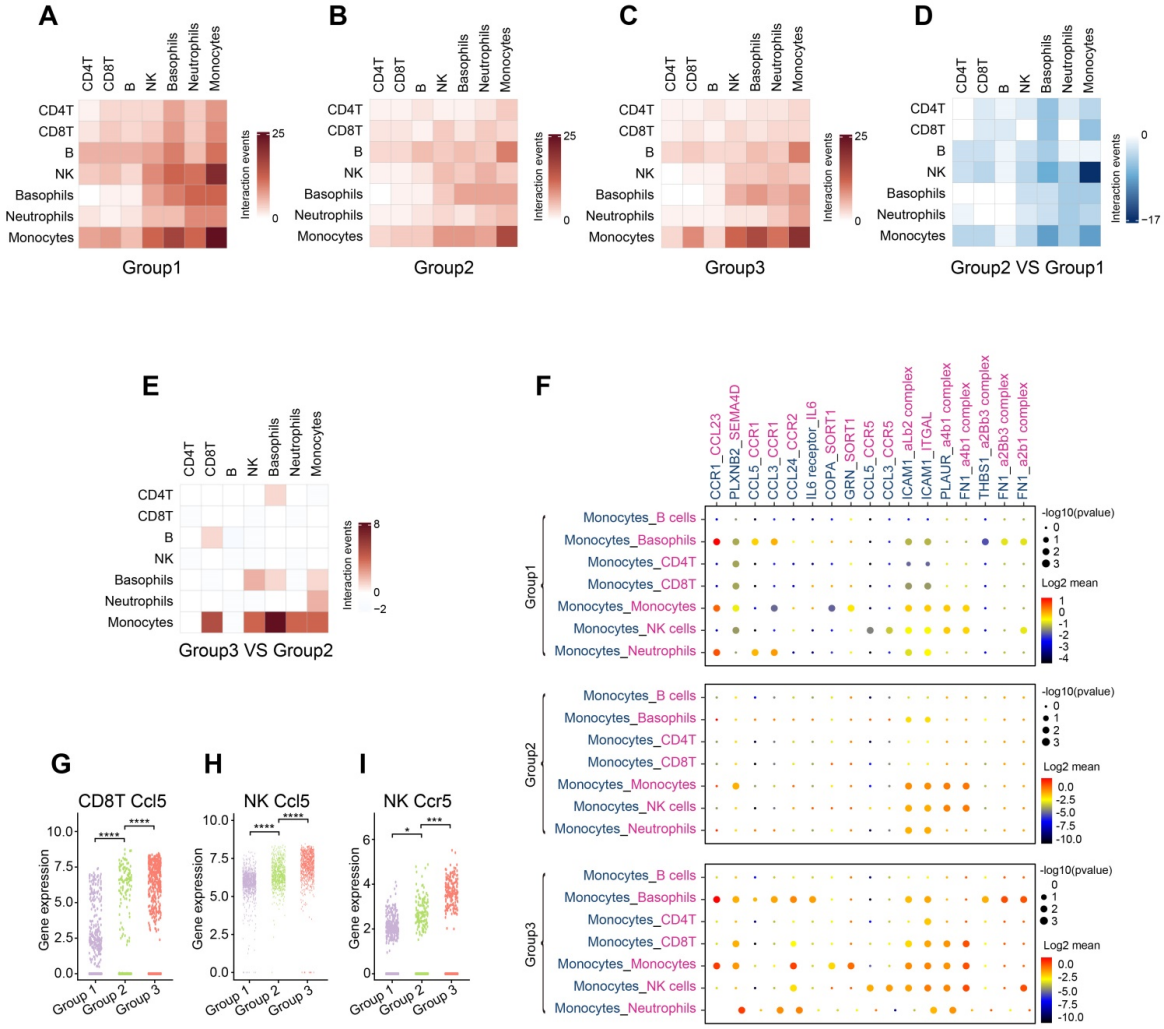
** Communication between immune cells.** (A-C) Heatmaps show the number of interaction events among each type of immune cell in group 1 (A), group 2 (B), and group 3 (C). (D) A heatmap representing the difference in interaction events among each type of immune cell between group 1 and group 2. (E) A heatmap representing the difference in interaction events among each type of immune cell between group 2 and group 3. (F) Cell-type-specific interactions between monocytes and the rest of the immune cell types where ligands were expressed on monocytes. Genes indicated in blue represent ligands, and those in red represent receptors. Circle sizes represent significance, which was defined as -log10 (p-value). Color bars from blue to red represent the normalized expression value of both ligands and receptors. (G-H) The expression level of Ccl5 in Cd8^+^ T cells (G) and NK cells (H) from the three experimental groups, respectively. (I) The expression level of Ccr5 in NK cells from three experimental groups.

Systemic immunity is a complex network in which immune cells interact with each other through ligand-receptor pairs. In this study, we applied CellPhoneDB, a well-established intercellular communication inference method, to determine how tumor burden and OH2 exerted impacts on the cell-cell interactions among peripheral immune cells. We noticed that in each experimental group, the cell-cell interactions among innate immune cells were tighter than those among adaptive immune cells (Figure 6A-C; Table S2-S4). With the increase in tumor burden, interactions among peripheral immune cells were significantly reduced, especially the interaction between NK cells and monocytes (Figure 6D). However, OH2 restored the interactions between monocytes and other immune cells, such as Cd8^+^T cells and NK cells (Figure 6E-F). We further investigated interactions between ligands on monocytes and receptors on Cd8^+^ T cells and found that after OH2 injection (group3 VS group2), monocytes began to interact with Cd8^+^ T cells through ICAM1 (Figure 6F). Previous studies suggested that ICAM1 expressed on antigen-presenting cells, such as monocytes, could promote T cell activation, proliferation, and cytokine production 27. In particular, our data showed that the ICAM1 expressed on monocytes interacted with ITGAL (also known as LFA-1) expressed on Cd8^+^ T cells after OH2 injection (Figure 6F), and this interaction played a key role in facilitating T cell activation 28, 29. In addition, the interaction between monocytes and NK cells mediated by FN1 was increased after OH2 injection (Figure 6F), which maintained the survival of mouse natural killer (NK) cells via the CD11b/Src/β-catenin pathway as demonstrated by a previous report 30. These results altogether indicated that monocytes positively regulated Cd8^+^ T cells and NK cells after OH2 injection. Since Ccl5 was the main cytokine upregulated in Cd8^+^ T cells and NK cells after OH2 injection (Figure 6G-H, Figure S2), we wondered which type of immune cell was mainly affected by Ccl5. As a result, we noticed that Ccr5, the most important receptor for Ccl5, was increased in NK cells. Thus, the Ccl5-Ccr5 axis was promoted by Cd8^+^ T cells-NK cells interactions and NK cells-NK cells interactions, which significantly enhance tumor infiltration and anti-tumor effects of NK cells 31.

### Effects of peripheral innate and adaptive immune responses induced by OH2 on distant tumor lesions

**Figure 7:**
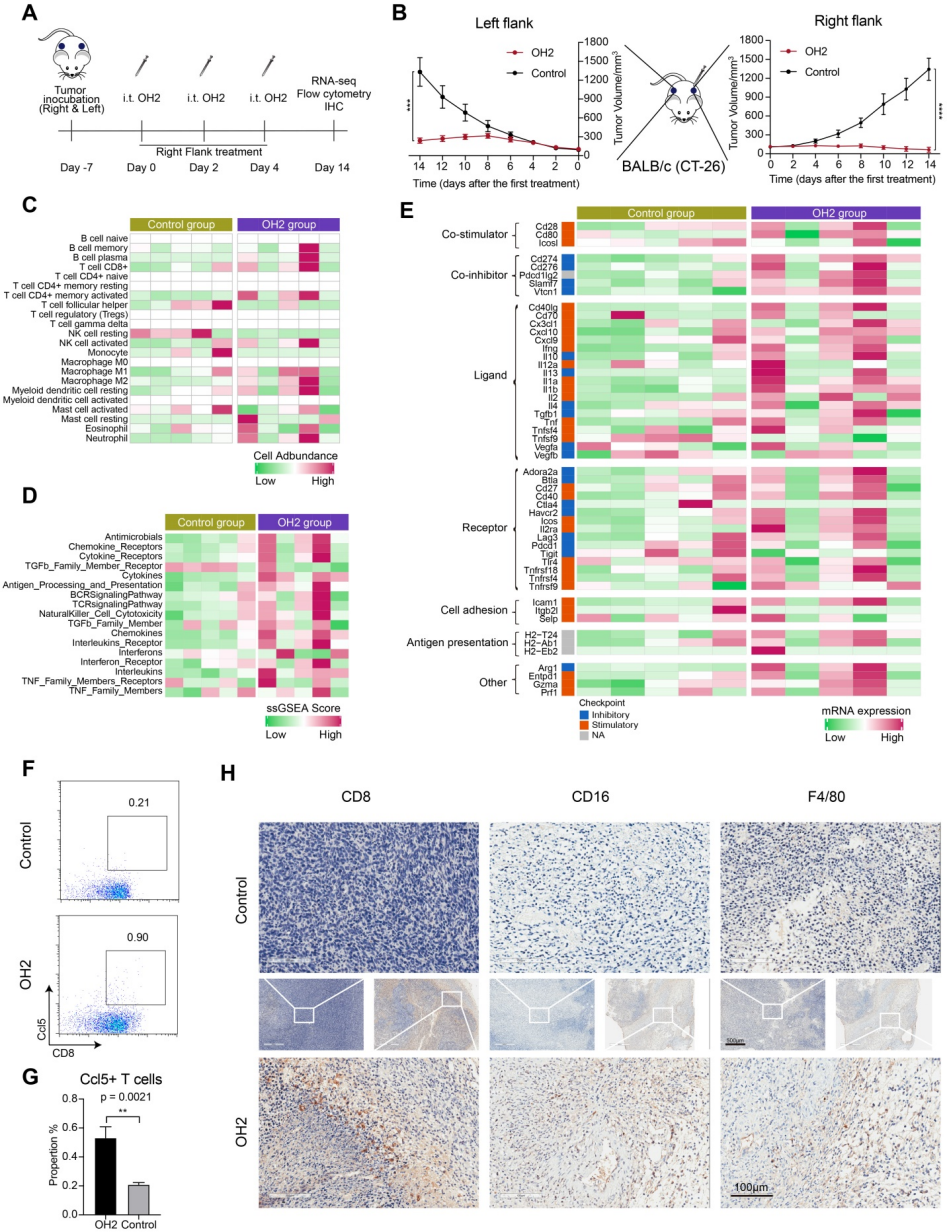
**Effects of OH2 on distant tumor lesions.** (A) Timeline of tumor injection and treatment. At each time point, only the right-side tumor was treated. i.t., intratumoral injection. (B) The Left and right panels show the growth curves for the left and right flank tumors from the two groups (PBS and OH2), respectively (n = 6). (C-E) A heatmap showing the infiltration level of 22 types of immune cells (C), the activation degree of 17 key immune pathways (D), and the scaled expression of key immunomodulators (E) in left flank tumors of the control group and the OH2 group. (F) Representative flow cytometric analysis results of Ccl5+CD8+ T cells. (G) The percentages of Ccl5+CD8+ T cells were determined 14 days after grouping. (H) Histological appearance of representative untreated flank tumor samples from each treatment group. Middle, original magnification, × 20. Top and bottom, original magnification, × 100.

Thus far, we in this study concluded that NK cells and Cd8^+^ T cells in the peripheral blood were activated by OH2. However, whether these cells could infiltrate into the tumor microenvironment to diminish distant tumor sites is still unknown. To answer this question, we designed and performed another animal experiment illustrated in Figure 7A. In this experiment, the immunocompetent BALB/c mice received inoculation of CT26 tumor cells on the right flank and left flank. The time that mice received tumor inoculation was referred to as day -7. Then these mice were equally divided into two groups: the OH2 group and the control group. In the OH2 group, OH2 was injected into the right flank tumor of each mouse on days 0, 2, and 4. In the control group, PBS was injected into the right flank tumor of each mouse on days 0, 2, and 4. Then we collected the left flank tumor (distant tumor) of each mouse on day 14 and performed downstream experiments. The data showed that unilateral (right) use of OH2 treatment not only inhibited the right flank tumors, but also suppressed the growth of left flank (distant) tumors (Figure 7B). Besides, as demonstrated in our previous study 32, after intratumorally injecting OH2 on mice bearing tumors from CT-26 cells, OH2 would only distribute in the injection site and not in blood and other organs. These results suggested that the therapeutic effects of OH2 on distant tumors may be mediated by systemic immunity. To interrogate whether the OH2-activated peripheral immune cells could infiltrate into the distant tumor lesions, we performed RNA-sequencing on the left flank tumors. As a result, the overall immune cell infiltration was promoted after OH2 injection into the right flank tumors. Particularly, the infiltration of Cd8^+^ T cells and activated NK cells was increased, while that of resting NK cells was decreased (Figure 7C). In addition, the activation degree of many immune pathways was also boosted within left flank tumors, such as the “TCR signaling pathway” and “NK cell cytotoxicity” (Figure 7D). We further evaluated the expression of key immune-related genes and noticed that the overall expression of known immunomodulators was promoted in left flank tumors after OH2 injection into right flank tumors (Figure 7E). All these results suggested that the immune microenvironment of left flank tumors was heated up by OH2, and the peripheral NK cells and Cd8^+^ T cells activated by OH2 could infiltrate into the left flank tumors to exert their anti-tumor effects. Flow cytometry and immunohistochemistry also confirmed these results. After OH2 treatment, the proportion of Ccl5^+^ CD8^+^ T cells in the periphery of mice was significantly increased (p = 0.0021) (Figure 7F and G). Moreover, the infiltration of CD8^+^ T cells, NK cells (CD16^+^), and mononuclear macrophages (F4/80^+^) in left flank tumors after OH2 therapy was significantly promoted (Figure 7H).

**Figure 8:**
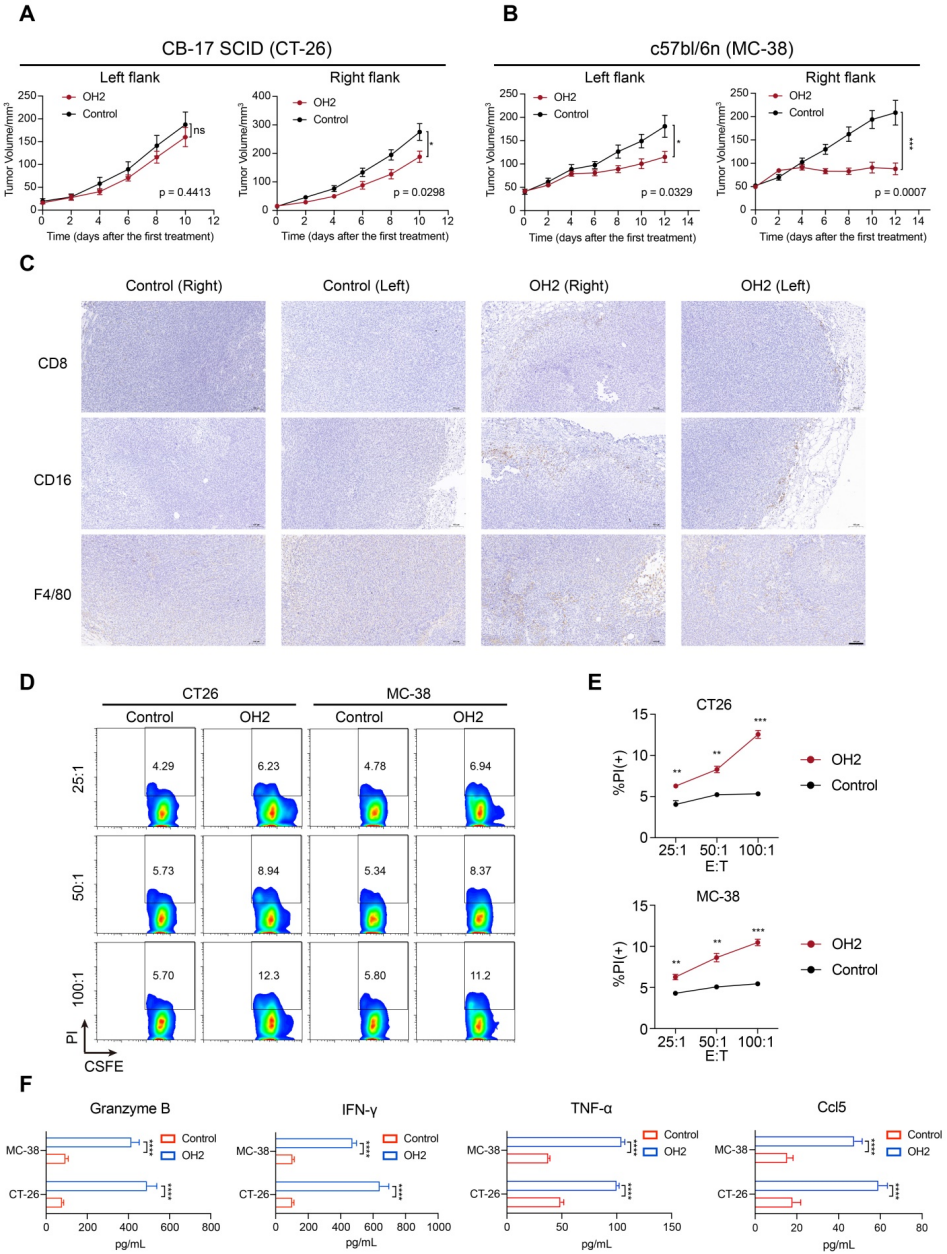
**OH2 enhanced anti-tumor systemic immunity in vivo**. (A) The left and right panels show the growth curves for the left and right flank tumors from the two groups (PBS and OH2) of CB-17 SCID mouse models, respectively (n = 8). (B) The left and right panels show the growth curves for the left and right flank tumors from the two groups (PBS and OH2) of c57bl/6n mouse models, respectively (n = 8). (C) Histological appearance of representative tumor samples from each group of c57bl/6n mouse models. Original magnification, × 100, scale bar, 100 μm. (D) Flow cytometric analysis results of the representative sample showing the killing functions of lymphocytes. (E) The killing functions of lymphocytes treated with different cancer cell lysates detected by CFSE/PI. Three E : T ratios were set for each cell line (25 : 1, 50 : 1, and 100 : 1). The data are averages from three samples per treatment group. **, p < 0.01, ***, p < 0.001. (F) Supernatant from the CTL assay was tested for Granzyme B, IFN-γ, TNF-α, and Ccl5 by ELISA. ****, p < 0.0001.

To further validate the anti-tumor effects of peripheral T and NK cells activated by OH2 on distant tumor lesions, we performed similar mouse experiments as shown in Figure 7A, except using the CB17 SCID mice that lack functional T lymphocytes but have normal NK cells. As a result, after injecting OH2 into the right flank tumor sites, tumor growth was significantly suppressed only in the treated (right) flank. In the untreated (left) flank, the therapeutic effect of OH2 was less pronounced (Figure 8A). Comparing these results with results from the immunocompetent BALB/c mice (Figure 7B), we could conclude that although activated NK cells have some anti-tumor effects, peripheral T cells activated by OH2 were indispensable for distant tumor regression.

To prove that the effects of OH2 could be generalized to different cell lines and mouse models, we also performed the OH2 treatment experiment using the immunocompetent C57bl/6n mice inoculated with MC-38 cell line. Consistent with results derived from the BALB/c mice inoculated with CT-26 cell line (Figure 7B), unilateral (right flank) injection with OH2 significantly inhibited the growth of treated (right) flank tumors as well as untreated (left) flank tumors (Figure 8B), and OH2 treatment also increased the infiltration of CD8+ T cells, NK cells (CD16+), and mononuclear macrophages (F4/80+) in both flanks (Figure 8C).

To further confirm that the peripheral immune system activated by OH2 was indeed anti-tumor immunity, splenic lymphocytes were isolated from the treatment group and the control group of the immunocompetent mouse models and co-cultured with CT26 cells or MC38 cells at an effector/target cell (E : T) ratio of 25 : 1, 50 : 1 and 100 : 1, respectively. Compared with the control group, the lymphocytes in the OH2 group showed a higher specific response to tumor cells (Figure 8D-E). ELISA showed that OH2 treatment significantly induced the expression of Granzyme B, IFN-γ, TNF-α, and Ccl5 in lymphocytes (Figure 8F).

**Figure 9:**
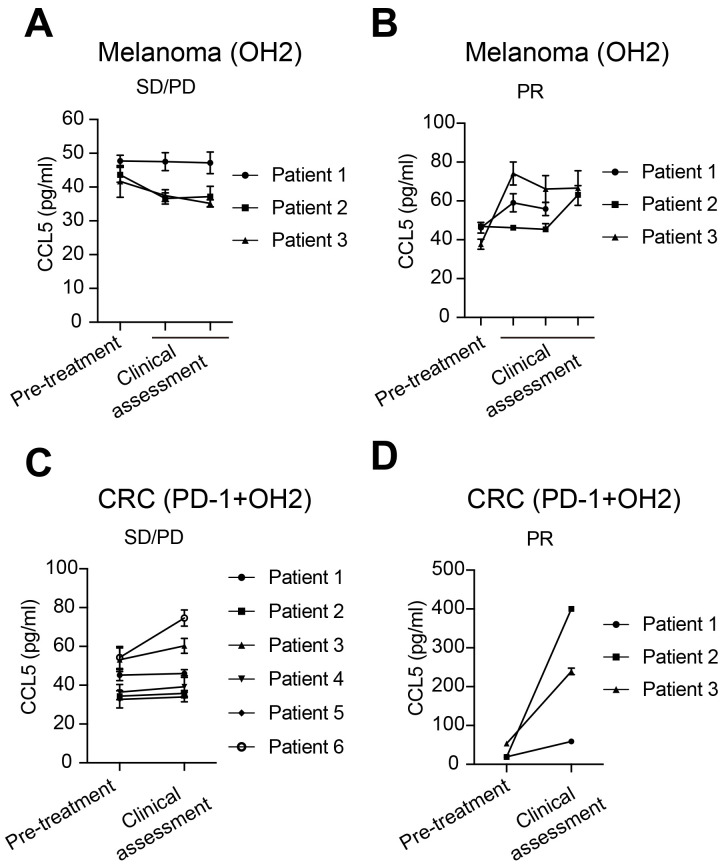
** Serum CCL5 level correlated with clinical response.** (A) CCL5 expression levels in melanoma patients with SD/PD receiving OH2 monotherapy. (B) CCL5 expression levels in melanoma patients with PR receiving OH2 monotherapy. (C) CCL5 expression levels in colorectal cancer patients with SD/PD receiving OH2 combined with PD-1 antibody. (D) CCL5 expression levels in colorectal cancer patients with PR receiving OH2 combined with PD-1 antibody.

Finally, in clinical samples, we evaluated the correlation between serum CCL5 levels and clinical responses to OH2 treatment. For melanoma patients treated with OH2 monotherapy, CCL5 levels remained unchanged or decreased in patients with stable disease (SD) and progressive disease (PD) (Figure 9A), while CCL5 levels elevated in patients having partial response (PR) (Figure 9B). For colorectal cancer patients treated with OH2 combined with PD-1 antibody, the CCL5 level of PR patients showed a more significant upward trend than SD/PD patients (Figure 9C-D), indicating that CCL5 is a potential candidate for the companion diagnostics for OH2 therapy.

## Discussion

Previous studies on oncolytic virus only focused on the effects of OV therapy on the TME and ignored the systemic immunity that is required to drive effective natural and therapeutically induced anti-tumor immune responses. To our knowledge, this study is the first to mainly characterize the perturbation of systemic immunity induced by OH2 based on single-cell sequencing. We demonstrated that OH2 therapy can induce systemic immune changes primarily manifested by the monocyte-mediated inflammation and the increased activation of NK cells and cytotoxic CD8^+^ T cells. In addition, these systemic immune alterations are effective mechanisms for OH2 therapy to suppress distant tumor lesions.

Recent studies have shown that the occurrence and development of tumors are accompanied by changes in the systemic immune landscape 33-37, and the increased tumor burden often directs systemic immunity into an inhibition state 13. Since OH2 could reduce the tumor burden, the overall effects of OH2 on systemic immunity came from the diminished tumor burden and the anti-tumor immune responses directly induced by OH2. Figuring out the effects of increased and decreased tumor burden on systemic immunity would help us determine how extensive were the anti-tumor immune responses directly induced by OH2. In this study, our results showed that elevated tumor burden led to an increased proportion of myeloid cells, especially neutrophils and monocytes, which was consistent with other reports 38.

Although OH2 therapy did not significantly change the ratio of myeloid cells to lymphocytes, it effectively restored the communication between immune cells, especially monocytes with CD8+ T cells and NK cells. Monocytes are a highly plastic population of innate immune cells 39 that function by regulating other immune cells, producing cytokines, and presenting antigens 40-42. In mice, monocytes can generally be divided into CX3CR1^hi^ (patrolling monocytes) and CX3CR1^low^ (inflammatory monocytes) subgroups according to different CX3CR1 expression levels 43. The two subgroups differ significantly in the expression of cytokines and cytokine receptors and their roles in inflammatory and pathological processes 41, 43, 44. Our results suggested that OH2 therapy promoted the proportion of inflammatory monocytes (also known as Tip-DCs) in peripheral blood, which are generally able to present antigens 45 and are necessary for the function of activated cytotoxic T lymphocytes 46. Interestingly, some reports have proven that patrolling monocytes can recruit large numbers of NK cells to tumor sites, especially metastatic tumor lesions, through the release of cytokines, such as CCL3, CCL4, and CCL5 47-49. Although our data suggested that OH2 treatment reduced the proportion of patrolling monocytes in peripheral blood, this may be partly due to the influx of patrolling monocytes into the tumor site. In general, monocytes are not just precursors of macrophages and dendritic cells but also promote the overall coordination of the immune system 50. Our data showed that, on the one hand, OH2 treatment can effectively enhance the association between monocytes and CD8+ T cells and NK cells; on the other hand, the increased infiltration of CD8+ T cells and NK cells in distant tumor sites may be the result of chemotactic recruitment by patrolling monocytes. This indicated that monocytes might be the core component coordinating systemic immunity during OH2 treatment.

Among all peripheral immune cell subtypes, the CD8^+^ T cells and mature NK cells were activated most significantly after OH2 treatment. CD8^+^ T cells have been the focus of cancer immunotherapy and have long been recognized as the most critical immune component for tumor eradication 51. Typically, CD8^+^ T cells kill tumor cells by secreting large amounts of cytotoxic molecules, such as granzymes and perforin, and cytokines, such as TNF-α and IFN-γ 52. Our data represented that OH2 treatment can boost the release of cytotoxic molecules and cytokines by cytotoxic CD8^+^ T cells, which are undoubtedly beneficial for tumor regression and the reinforcement of anti-tumor immune responses. NK cells recognize tumor cells and eliminate them quickly through unique mechanisms 53. Existing studies have shown that NK cells not only kill tumor cells directly through their cytotoxicity but also regulate the activity of other immune cells by secreting a variety of cytokines and chemokines 53, 54. Both cytotoxic NK cells and cytokine-secreting NK cells were activated by OH2 and definitely played a positive role in amplifying the anti-tumor immune responses.

In addition, one interesting alteration in the systemic immunity induced by OH2 treatment is the significantly promoted expression of Ccl5. Ccl5 plays a critical role in coordinating the peripheral immune cells. It has been reported that Ccl5 can induce the infiltration of CD8^+^ T cells 55, CD4^+^ T cells 56, and NK cells 31 into the TME, thereby enhancing the anti-tumor immune responses. Bottcher et al. also reported that Ccl5 secreted by NK cells plays a key role in activating dendritic cells and recruiting these cells into the TME 57. Therefore, we speculated that Ccl5 might play an important role in the response to OH2 treatment and may be a potential molecular marker worthy of further exploration.

Although immune biomarkers are of great clinical value for the guidance and decision-making of immunotherapy, there is no complete study on establishing systemic immune biomarkers, especially for OV treatment 9. Our data comprehensively demonstrated alterations in the systemic immunity induced by OH2 treatment. Among them, Ccl5 has the potential to predict treatment efficacy and may be used as a circulating protein biomarker of companion diagnostics for OH2 treatment. In addition, changes in the proportion and function of CD8^+^ T cells, NK cells, and monocytes may also be associated with efficacy and are potential peripheral cell biomarkers. These possibilities are being validated by ongoing OH2 clinical trials conducted by us.

In conclusion, this study is the first to comprehensively characterize the changes in the systemic immune landscape induced by OV therapy using single-cell sequencing. Our data suggested that OH2 therapy can effectively activate systemic immunity across the board and induced sustained anti-tumor immune responses. Further analyses revealed that the interaction of monocytes with T cells and NK cells was critical for systemic immune remodeling and activation. These findings not only shed new light on the anti-tumor mechanisms of OH2, but also provides useful guidance for the establishment of molecular markers of companion diagnostics for OH2 treatment and the improvement of oncolytic therapy strategies.

## Supplementary Material

Supplementary figures.

Supplementary table 1.

Supplementary table 2.

Supplementary table 3.

Supplementary table 4.

## Abbreviations

OV: Oncolytic virus
HSV: Herpes simplex virus
T-VEC: Talimogene Laherparepvec
oHSV1: Oncolytic herpes simplex virus type 1
TME: The tumor microenvironment
CTL: Cytotoxic T lymphocyte
OH2: Oncolytic virus HSV-2
scRNA-seq: Single-cell RNA sequencing
s.c.: Subcutaneous injection
i.t.: Intratumoral injection
PCA: Principal component analysis
DEGs: Differentially expressed genes
GO: Gene Ontology
AUC: Area-under-the-curve
IHC: Immunohistochemistry
pDCs: Plasmacytoid dendritic cells
SD: Stable disease
PD: Progressive disease
PR: Partial response
