# Supplementary material for: Single-cell transcriptomics of peripheral blood reveals anti-tumor systemic immunity induced by oncolytic virotherapy
Source: Theranostics. 2022 Oct 17;12(17):7371–89. doi: 10.7150/thno.74075 (PMC9691353; doi:10.7150/thno.74075)
Supplement: Supplementary file 1 — Supplementary figures. [file thnov12p7371s1.pdf]

Figure S1

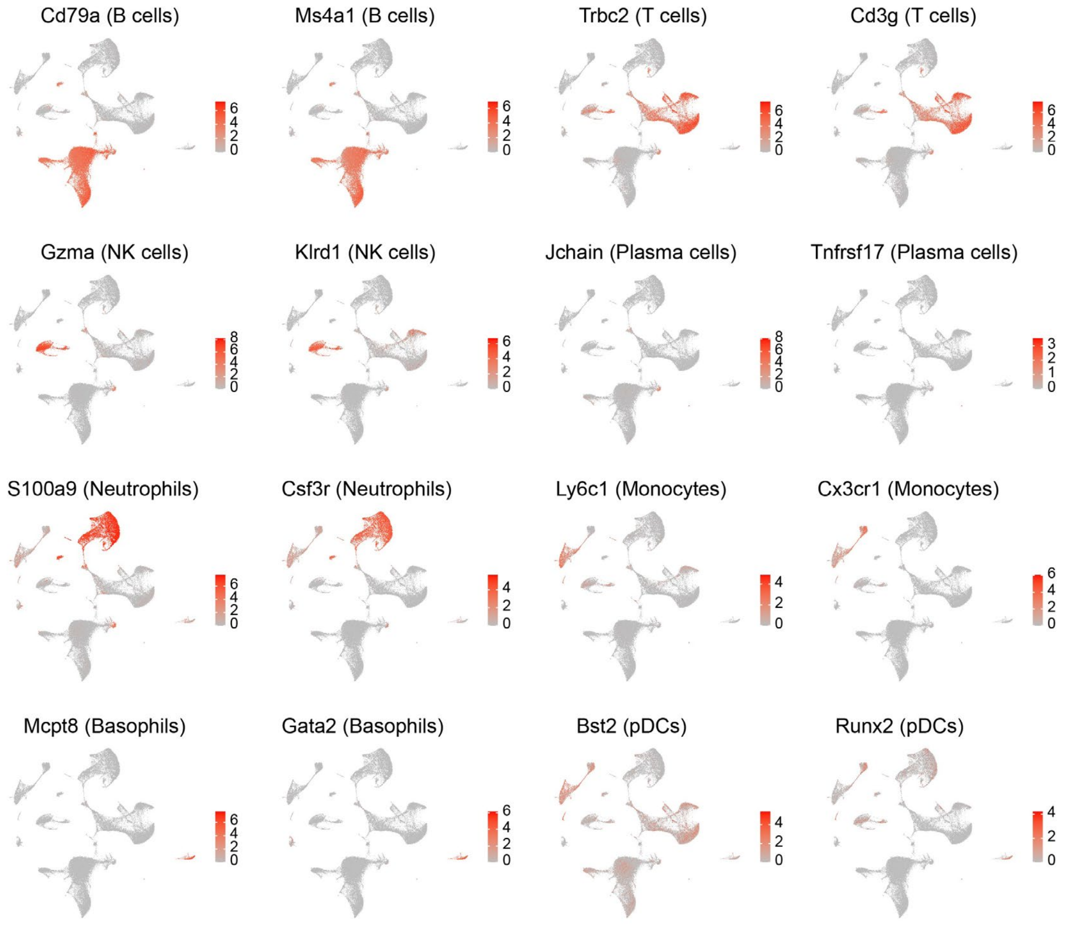

Fig. S1: Single cell gene expression for representative immune cell type-enriched genes.

Figure S2

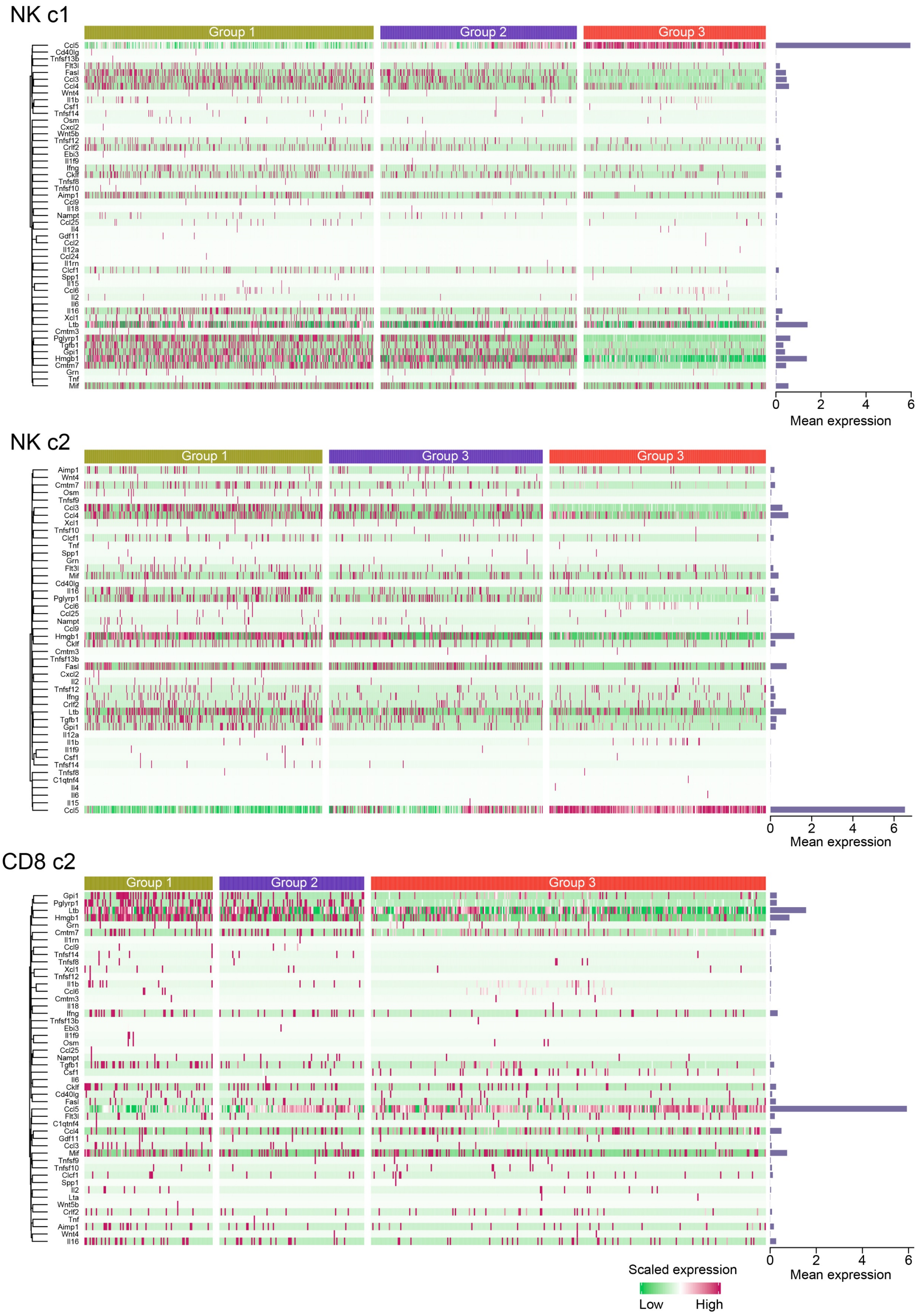

Fig.S2: Heatmaps showing the levels of cytokines produced by NK c1, NK c2, and CD8 c2. Bars represent the mean expression of each cytokine.
